# The Modulatory Effect of Ellagic Acid and Rosmarinic Acid on Ultraviolet-B-Induced Cytokine/Chemokine Gene Expression in Skin Keratinocyte (HaCaT) Cells

**DOI:** 10.1155/2014/346793

**Published:** 2014-08-04

**Authors:** Serena Lembo, Anna Balato, Roberta Di Caprio, Teresa Cirillo, Valentina Giannini, Franco Gasparri, Giuseppe Monfrecola

**Affiliations:** ^1^Section of Dermatology, Department of Clinical Medicine and Surgery, University of Naples Federico II, Via S. Pansini 5, 80131 Naples, Italy; ^2^Rottapharm Madaus Dermo-Cosmetic R&D Division, Via Valosa di Sopra 9, 20052 Monza, Italy

## Abstract

Ultraviolet radiation (UV) induces an increase in multiple cutaneous inflammatory mediators. Ellagic acid (EA) and rosmarinic acid (RA) are natural anti-inflammatory and immunomodulatory compounds found in many plants, fruits, and nuts. We assessed the ability of EA and RA to modulate IL-1*β*, IL-6, IL-8, IL-10, MCP-1, and TNF-*α* gene expression in HaCaT cells after UVB irradiation. Cells were treated with UVB (100 mJ/cm^2^) and simultaneously with EA (5 *μ*M in 0.1% DMSO) or RA (2.7 *μ*M in 0.5% DMSO). Moreover, these substances were added to the UVB-irradiated cells 1 h or 6 h before harvesting, depending on the established UVB-induced cytokine expression peak. Cytokine gene expression was examined using quantitative real time polymerase chain reaction. RA produced a significant reduction in UVB-induced expression of IL-6, IL-8, MCP-1, and TNF-*α* when applied at the same time as irradiation. EA showed milder effects compared with RA, except for TNF-*α*. Both substances decreased IL-6 expression, also when applied 5 h after irradiation, and always produced a significant increase in UVB-induced IL-10 expression. Our findings suggest that EA and RA are able to prevent and/or limit the UVB-induced inflammatory cascade, through a reduction in proinflammatory mediators and the enhancement of IL-10, with its protective function.

## 1. Introduction

Ultraviolet radiation (UV) induces cutaneous inflammatory reactions characterized by an increase in many cytokines, such as interleukin (IL)-1*β*, IL-6, IL-8, IL-10, monocyte chemoattractant protein (MCP)-1, and tumour necrosis factor alpha (TNF-*α*) [1].

Ellagic acid (EA) is a polyphenol present in fruits, such as grapes, strawberries, pomegranates, and walnuts, as well as in several medicinal plants, and is known to exert multiple biological actions, including inhibition of proliferation, angiogenesis, oxidation, and other processes involved in inflammation and carcinogenesis [2–4].

Rosmarinic acid (RA) is a naturally occurring hydroxylated compound widely distributed in Labiatae herbs, which include rosemary, sweet basil, and perilla. Similar to EA, RA exhibits many bioactivities, mainly antioxidant and anti-inflammatory in nature, and also antiviral and antibacterial activities [5, 6]. Its mechanism of action is linked to inhibition of lipoxygenases and cyclooxygenases and to interference with the complement cascade. In addition, RA, by inhibiting phospholipase C-gamma 1 and IL-2-inducible T-cell kinase, is able to regulate Ca^2+^-dependent pathways involved in T-cell receptor signaling [5–8].

Both EA and RA have been shown to be valuable defences against noxious substances, whether they are toxic chemicals or ultraviolet radiations that affect our skin daily.

EA is able to prevent collagen degradation by blocking matrix metalloproteinase production in UV-B-exposed fibroblasts and to diminish production of proinflammatory cytokines and skin infiltrating macrophages [3]. Hence, the photoprotective effects of EA are linked to decreased skin inflammation and wrinkle formation [3]. RA exerts its photoprotective activity through free radical scavenging and stimulation of melanin production [7]. Moreover, a mitigating effect of RA on cutaneous inflammatory diseases, such as atopic dermatitis (AD), has been demonstrated [8].

In this* in vitro* study, we investigated the ability of EA and RA to modulate the effects of UVB irradiation in immortalized keratinocytes.

## 2. Materials and Methods

### 2.1. Cell Culture

Spontaneous immortalized keratinocytes (HaCaT cells) were grown in Dulbecco's modified Eagle's medium (DMEM, GIBCO, Grand Island, NY) containing 10% fetal bovine serum (FBS, GIBCO, Grand Island, NY), 2 mM L-glutamine (GIBCO, Grand Island, NY), and antibiotics (100 IU/mL penicillin G, 100 *μ*g/mL streptomycin, GIBCO, Grand Island, NY). Cells were cultured in a humidified incubator at 37°C with 5% CO_2_, until they reached a confluence of about 80%.

### 2.2. UVB Irradiation

As the UVB source, Philips TL12/60W fluorescent lamps (Philips, Eindhoven, The Netherlands), emitting UVB light between 290 and 320 nm, with a peak emission of 300 nm, were used. The intensity of UVB irradiation, measured with a UV meter (Spectrolyne mod., Spectronics Corp., Westbury, NY, USA) was 0.8 mW/cm^2^. The optimal dose of UVB 100 mJ/cm² was determined by irradiating HaCaT as previously described [9]. Prior to UVB irradiation, HaCaT cells were properly washed with phosphate buffered saline solution (PBS) and covered with a thin layer of PBS which, immediately after irradiation, was removed and replaced with DMEM.

### 2.3. Chemicals

EA and RA were supplied by Rottapharm Madaus Dermo-Cosmetic R & D Division (Monza, Italy). Multiple concentrations (2.5; 5; 10 *μ*M for EA and 2.7; 13.7; 27.5; 55 *μ*M for RA) were prepared in dimethylsulfoxide (DMSO, Sigma-Aldrich, St. Louis, MO, USA) and diluted in DMEM so that the final concentration of DMSO in the medium was 0.1% for EA and 0.5% for RA.

### 2.4. Determination of Cell Viability

Cell viability was assessed using trypan blue stain, after treating HaCaT with the above-mentioned concentrations of EA and RA, UVB 100 mJ/cm², or after a combination of UVB and substances for 24 h.

### 2.5. Study Design of UVB Irradiation and EA/RA Incubation Times

The time for peak expression of IL-1*β*, IL-6, IL-8, IL-10, MCP-1, and TNF-*α* was determined by gene analysis in a UVB-irradiated versus unirradiated control at 6, 12, and 24 h: IL-1*β*, IL-6, and IL-8 mRNA expression was mainly induced 6 h after UVB irradiation, whereas peak expression of IL-10, MCP-1, and TNF-*α* mRNA was recorded 24 h after UVB exposure [9].

**Figure 1:**
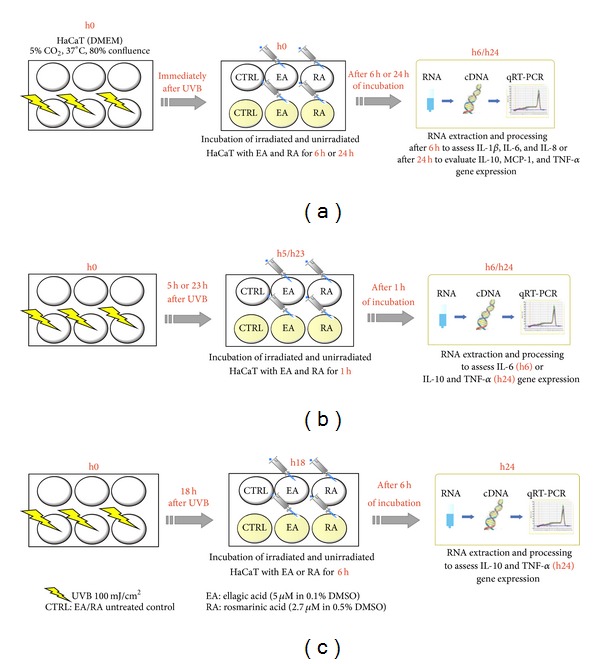
Study design. Schematic representation of the experimental settings with UVB irradiation and EA/RA incubation times, as described in Section 2.5.

The ability of EA and RA to modulate expression of the above inflammatory mediators in HaCaT cells after UVB irradiation was assessed at different time points. As a first step, immediately after UVB irradiation (100 mJ/cm²), HaCaT cells were separately incubated with EA (5 *μ*M in 0.1% DMSO) or RA (2.7 *μ*M in 0.5% DMSO) for 6 h and 24 h. mRNA was extracted at 6 and 24 h, respectively, in line with each cytokine expression peak (Figure 1(a)). As a second step, 5 h or 23 h after irradiation (i.e., 1 h before peak cytokine expression was reached after UVB exposure), HaCaT cells were incubated with EA or RA (Figure 1(b)). Lastly, in order to further assess IL-10 and TNF-*α*, cells were incubated 18 h after UVB exposure with EA or RA (i.e., 6 h before peak cytokine expression) and mRNA was extracted at 24 h (Figure 1(c)).

### 2.6. mRNA Extraction and Reverse Transcription to cDNA for qRT-PCR

Total mRNA was isolated using the RNeasy Mini Kit (Qiagen, Doncaster, Australia) according to the manufacturer's instructions. cDNA was prepared using the Transcriptor High Fidelity cDNA Synthesis Kit (Roche, Indianapolis, IN, USA). Quantitative real time polymerase chain reaction (qRT-PCR; LightCycler, Roche, Indianapolis, IN) was performed to confirm differences in the expression levels of IL-1*β*, IL-6, IL-8, IL-10, MCP-1, and TNF-*α*. The amount of mRNA for a given gene in each sample was normalized to the amount of mRNA of 18S reference gene in the same sample. Fold induction of gene expression was calculated using the ΔΔCt method, as described previously [10]. The primer sequences were as follows:

 IL-1*β* forward: 5′-TCC TGC GTG TTG AAA GAT GAT AA-3′
 IL-1*β* reverse: 5′-CAA ATC GCT TTT CCA TCT TCT TC-3′
 IL-6 forward: 5′-TAC CCC CAG GAG AAG ATT CC-3′
 IL-6 reverse: 5′-GCC ATC TTT GGA AGG TTC AG-3′
 IL-8 forward: 5′-AGA CAG CAG AGC ACA CAA GC-3′
 IL-8 reverse: 5′-ATG GTT CCT TCC GGT GGT-3′
 IL-10 forward: 5′-TGA GAA CAG CTG CAC CCA CTT-3′
 IL-10 reverse: 5′-ATC TCC GAG ATG CCT TCA GC-3′
 MCP-1 forward: 5′-CCA GCA TGA AAG TCT CTG CC-3′
 MCP-1 reverse: 5′-ATA ACA GCA GGT GAC TGG GG-3′
 TNF-*α* forward: 5′-CTG CTG CAC TTT GGA GTG AT-3′
 TNF-*α* reverse: 5′-AGA TGA TCT GAC TGC CTG GG-3′
 18S forward: 5′-AAC CCG TTG AAC CCC ATT-3′
 18S reverse: 5′-CCA TCC AAT CGG TAG TAG CG-3′.

Cycling conditions were as follows: denaturation (95°C for 10 min), amplification and quantitation (95°C for 10 s, 60°C for 5 s—45 s for IL-10—and 72°C for 10 s) repeated 40 times (38 times for IL-10 and 30 times for 18S), melting curve program (65–95°C with a heating rate of 0.1°C/s and continuous fluorescence measurement), and cooling step (40°C for 30 s).

### 2.7. Data Analysis

All statistical analyses were performed using GraphPad Prism 4.0 (GraphPad Software Inc, La Jolla, CA). Data that passed the normality test were analysed with a two-tailed *t*-test, otherwise with Wilcoxon. Values of *P* < 0.05 were considered significant. Data are expressed as means ± SD of three independent experiments, each performed in duplicate.

## 3. Results

### 3.1. Effect of EA and RA on Cell Viability

**Figure 2:**
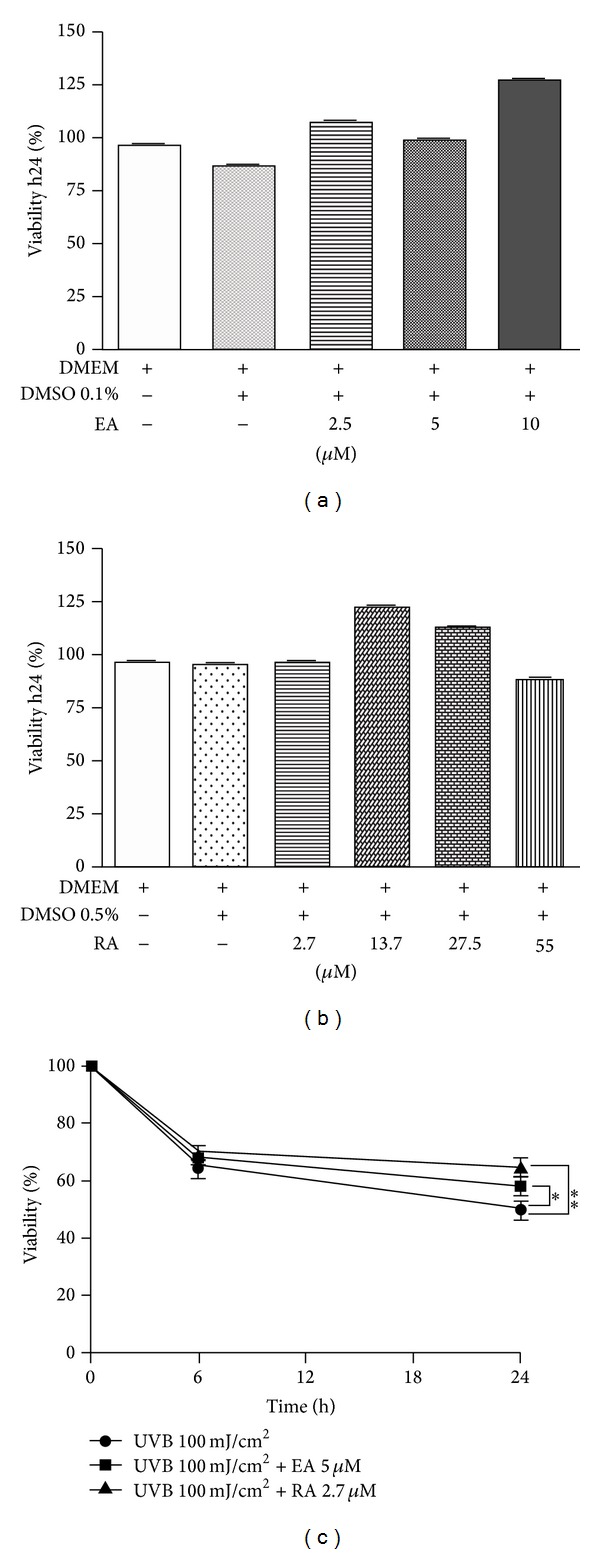
Evaluation of cell viability after 24 h incubation with EA or RA in UVB-irradiated and unirradiated cells. (a) EA (concentrations: 2.5; 5; 10 *μ*M); (b) RA (concentrations: 2.7; 13.7; 27.5; 55 *μ*M); (c) EA (5 *μ*M in 0.1% DMSO) or RA (2.7 *μ*M in 0.1% DMSO) applied immediately after UVB irradiation (100 mJ/cm²). Cell viability was measured using trypan blue. Statistical significance was determined with respect to the viability of UVB-irradiated cells (*P < 0.05; **P < 0.01).

None of the tested concentrations of EA and RA, when used alone, appreciably affected cell viability (Figures 2(a) and 2(b)); thereafter, EA 5 *μ*M in 0.1% DMSO and RA 2.7 *μ*M in 0.5% DMSO were used in our experimental settings, since, from preliminary tests, cell viability was very similar to the control (Figures 2(a) and 2(b)). When incubated for 24 h after UVB irradiation, RA was able to decrease UVB-induced mortality, increasing the cell viability rate from 50% to 65% (Figure 2(c)); this effect was milder for EA-treated cells (cell viability increased from 50% to 58%; Figure 2(c)).

### 3.2. EA/RA Modulation of Cytokine Expression When Incubated Immediately after UVB Irradiation

**Figure 3:**
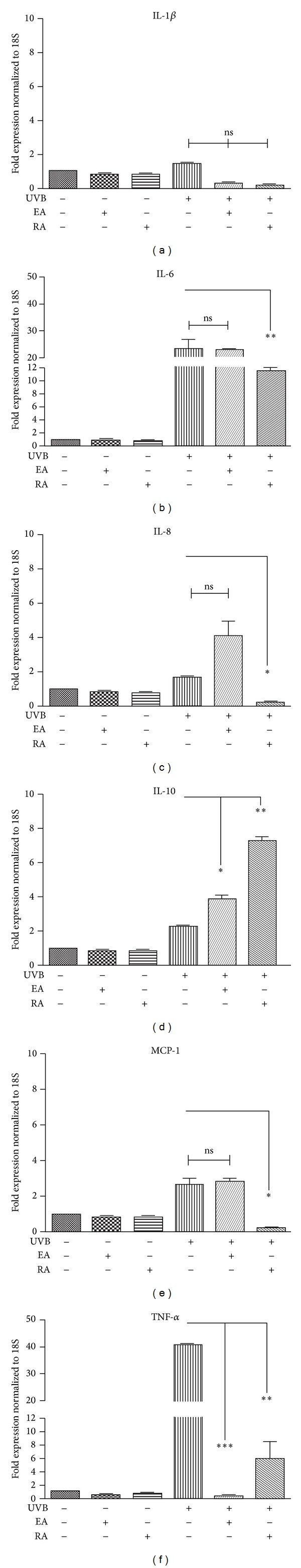
Assessment of cytokine gene expression in HaCaT cells incubated with EA (5 *μ*M in 0.1% DMSO) or RA (2.75 *μ*M in 0.5% DMSO) immediately after UVB exposure (100 mJ/cm²), through qRT-PCR. ((a), (b), and (c)) IL-1*β*, IL-6, and IL-8 gene expression in cells incubated immediately after UVB irradiation with EA or RA for 6 h; ((d), (e), and (f)) IL-10, MCP-1, and TNF-*α* gene expression in cells incubated immediately after UVB irradiation with EA or RA for 24 h. (*P < 0.05, **P < 0.01, and ***P < 0.001; ns: not statistically significant).

When incubated immediately after UVB irradiation, only RA was able to produce a statistically significant reduction in the UVB-induced increase in mRNA expression for IL-6, IL-8, and MCP-1 (Figures 3(b), 3(c), and 3(e)). In particular, RA was found to halve the 23-fold increase in IL-6 gene expression induced by UVB (*P* < 0.01). In relation to IL-8, EA induced a slight, nonstatistically significant increase in UV-induced gene expression. On the other hand, the 40-fold increase in TNF-*α* mRNA expression induced by UVB alone was strongly downregulated by RA (*P* < 0.01) and nearly reset by EA (*P* < 0.001, Figure 3(f)). Remarkably, the presence of EA and RA doubled and quadrupled, respectively, the 2-fold increase in UVB-induced IL-10 gene expression (Figure 3(d)). IL-1*β* expression after UVB was not affected either by EA or RA (Figure 3(a)).

### 3.3. EA/RA Modulation of Cytokine Expression When Incubated 1 h or 6 h before Peak Cytokine Expression Was Reached after UVB

**Figure 4:**
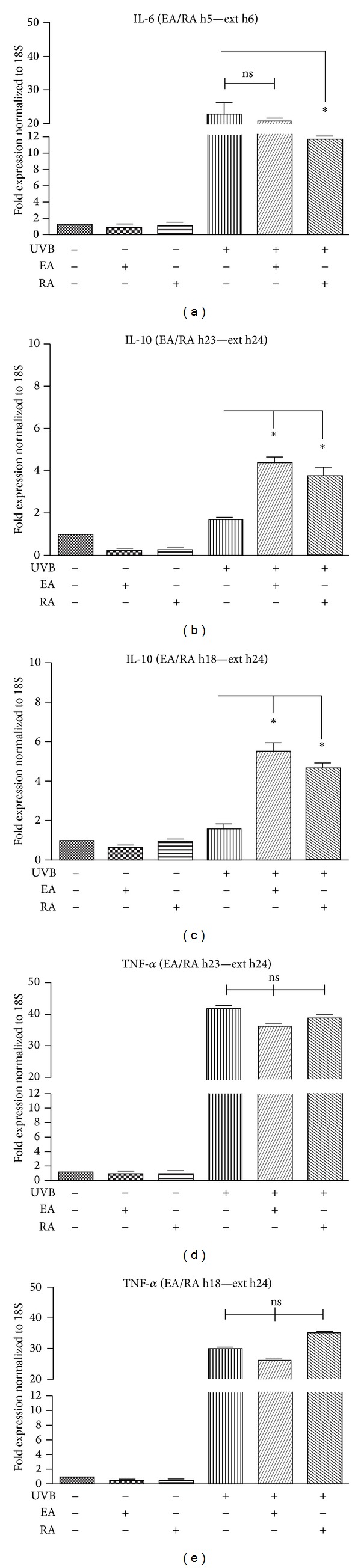
Assessment of IL-6, IL-10, and TNF-*α* gene expression in HaCaT cells irradiated with UVB (100 mJ/cm^2^) and incubated with EA (5 *μ*Min 0.1% DMSO) or RA (2.75 *μ*Min 0.5% DMSO), through qRT-PCR. (a) IL-6 gene expression in cells incubated, 5 h after UVB, with EA or RA for 1 h (i.e., 1 h before peak IL-6 expression was reached after UVB exposure). mRNA was extracted 6 h after UVB exposure (Ext h6); ((b), (d)) IL-10 and TNF-*α* gene expression in cells incubated, 23 h after UVB, with EA or RA for 1 h (i.e., 1 h before peak cytokine expression was reached after UVB exposure). mRNA was extracted 24 h after UVB exposure (Ext h24); ((c), (e)) IL-10 and TNF-*α* gene expression in cells incubated, 18 h after UVB, with EA or RA for 6 h (i.e., 6 h before peak cytokine expression). mRNA was extracted 24 h after UVB exposure (Ext h24). (*P < 0.05; ns: not statistically significant).

When the substances were incubated 5 h after irradiation (1 h before peak IL-6 expression), the expression of IL-6 induced by UVB showed a significant reduction following RA (Figure 4(a)). Both EA and RA induced a significant increase in UVB-induced IL-10 gene expression when applied 23 h after UVB exposure (1 h before peak IL-10 expression) (Figure 4(b)); earlier incubation of these substances, 18 h after irradiation (6 h before peak IL-10 expression), was slightly more effective (Figure 4(c)). In relation to TNF-*α* expression, neither EA nor RA seemed to be able to induce a significant reduction when added 23 or 18 h after UVB (Figures 4(d) and 4(e)).

## 4. Discussion

In this study, using immortalized human keratinocytes, we proved that EA and RA are able to modulate the expression of inflammatory mediators, such as TNF-*α*, IL-6, IL-8, MCP-1, and IL-10, in accordance with previous studies illustrating beneficial properties of EA and RA [2–8, 11, 12]. Using UVB and different EA and RA incubation times, we assessed the ability of the tested substances to prevent and limit irradiation-induced inflammation. Overall, these substances proved more effective after prolonged cell contact. When added 18 h or 23 h after irradiation, both EA and RA were unable to downregulate UVB-induced TNF-*α* expression. TNF-*α* is involved in the initiation and promotion of the inflammatory pathway following UVB: it exerts pleiotropic effects, including modulation of cell adhesion molecules, promotion of apoptosis, and activation of lymphocytes [1]. Hence, a 1 h or 6 h period of incubation with EA or RA is probably an inadequate time to reverse an already established inflammatory status. Indeed, EA and RA incubation for 24 h immediately after UVB irradiation were able to induce an appreciable reduction in TNF-*α* expression.

We found that EA and, to a more efficient extent, RA decreased expression of IL-6, a multifunctional cytokine induced in the early reaction to UVB. This corroborates the findings of Vostálová et al. [6], who demonstrated that RA reduced IL-6 secretion from UVB-irradiated HaCaT culture. Moreover, like other antioxidant compounds such as vitamin C [13], RA but not EA was able to downregulate the moderate increase in IL-8 and MCP-1 gene expression in UVB-irradiated keratinocytes. Indeed, following UVB-irradiation, IL-8 is upregulated in human keratinocytes and participates in the inflammatory process, stimulating neutrophil migration [13]. MCP-1, a member of the IL-8 supergene family, plays a critical role in the recruitment of monocytes and lymphocytes during the inflammatory response but, unlike IL-8, only a few studies have investigated the association between MCP-1 and UVB-induced inflammation [13].

With regard to IL-1*β* expression, EA and RA did not significantly affect the moderate increase in UVB-induced IL-1*β* expression. IL-1*β* is involved in inflammatory pathways triggered by many noxious stimuli such as infections or UVB irradiation [14]. Only one study demonstrated that topical application of EA diminished IL-1*β* production in hairless mice after UVB [3].* In vivo* models are probably more reliable for studying IL-1*β* after UV stimulation.

On the other hand, IL-10 gene expression after UVB seemed to be potentiated by both EA and RA. In particular, RA seemed to be more effective after a prolonged incubation period (24 h), inducing the highest IL-10 increase when added immediately after irradiation. EA produced a milder effect compared with RA after incubation for 1, 6, or 24 hours. IL-10 is a cytokine expressed by most T cells including regulatory T cells, antigen-presenting cells, macrophages, and epithelial cells. In recent years, it has been demonstrated that IL-10, which has traditionally been thought to contribute to the immunosuppressive milieu, is required for efficient immunosurveillance of the initiation and progression of skin tumours. More specifically, IL-10 induces infiltration and activation of intratumoural-specific cytotoxic CD8 + T cells, expressing interferon-*γ* and granzymes [15]. Furthermore, IL-10 has a recognised role in inflammatory skin conditions: it is able to depress contact hypersensitivity reactions but seems to be downregulated in AD patients during stress-related exacerbations [16, 17]. In light of this, our experimental results could explain and support the clinical observation made by Lee et al. [11]. They noticed an improvement in the signs and symptoms of AD after topical application of RA emulsion and explained the anti-inflammatory efficacy of RA through a reduction in TNF-*α* [11, 12]. Indeed, following our experiments, a direct effect of RA, and of EA, on the IL-10 pathway can also be hypothesized, in accordance with a recent study by El-Shitany et al. [18], who found a protective effect of EA against carrageenan-induced acute inflammation through enhancement of IL-10 in rats. The inhibition exerted by the two substances on the mitogen-activated protein kinases, which, in turn, are among the factors responsible for IL-10 downregulation [19–21], could explain the modulation of IL-10 expression. The fact that EA and RA are able to upregulate IL-10 and downregulate TNF-*α* could be explained by the different pathways activated by common cellular targets involved in the inflammatory cascade. For example, nuclear factor kappa B (NF-*κ*B), which is required for transcription of many proinflammatory mediators such as TNF-*α* [22], is inhibited by EA and RA [12, 18, 23]. Interestingly, IL-10 is involved in NF-*κ*B inhibition [24]; hence this could represent a link between the two mediator pathways. Nonetheless, since HaCaT keratinocytes do not express functional IL-10 receptors [25], the downregulation of TNF-*α* exerted by EA and RA in our experiments seems to have an independent pattern, unrelated to IL-10.

## 5. Conclusion

Our study reinforces the idea that EA and RA have multifaceted properties, including the ability to prevent and/or limit UVB-induced damage, increasing cell viability and decreasing the inflammatory response, through a reduction in multiple proinflammatory mediators and enhancement of the protective IL-10.
